# Serologic and PCR testing of persons with chronic fatigue syndrome in the United States shows no association with xenotropic or polytropic murine leukemia virus-related viruses

**DOI:** 10.1186/1742-4690-8-12

**Published:** 2011-02-22

**Authors:** Brent C Satterfield, Rebecca A Garcia, Hongwei Jia, Shaohua Tang, HaoQiang Zheng, William M Switzer

**Affiliations:** 1 Cooperative Diagnostics, LLC, Greenwood, SC 29646, USA; 2 Laboratory Branch, Division of HIV/AIDS Prevention, National Center for HIV/AIDS, Viral Hepatitis, STD, and TB Prevention, Centers for Disease Control and Prevention, Atlanta, GA 30333, USA

## Abstract

In 2009, a newly discovered human retrovirus, xenotropic murine leukemia virus (MuLV)-related virus (XMRV), was reported by Lombardi *et al*. in 67% of persons from the US with chronic fatigue syndrome (CFS) by PCR detection of *gag *sequences. Although six subsequent studies have been negative for XMRV, CFS was defined more broadly using only the CDC or Oxford criteria and samples from the US were limited in geographic diversity, both potentially reducing the chances of identifying XMRV positive CFS cases. A seventh study recently found polytropic MuLV sequences, but not XMRV, in a high proportion of persons with CFS. Here we tested blood specimens from 45 CFS cases and 42 persons without CFS from over 20 states in the United States for both XMRV and MuLV. The CFS patients all had a minimum of 6 months of post-exertional malaise and a high degree of disability, the same key symptoms described in the Lombardi *et al. *study. Using highly sensitive and generic DNA and RNA PCR tests, and a new Western blot assay employing purified whole XMRV as antigen, we found no evidence of XMRV or MuLV in all 45 CFS cases and in the 42 persons without CFS. Our findings, together with previous negative reports, do not suggest an association of XMRV or MuLV in the majority of CFS cases.

## Findings

The xenotropic murine leukemia virus (MuLV)-related virus (XMRV) is a retrovirus capable of infecting human cell lines and was recently found in some persons with prostate cancer [1]. Conflicting reports of XMRV in Europe and the US show XMRV prevalence between 0 and 27% in prostate cancer patients [2-4]. More recently, Lombardi *et al*. reported finding XMRV in 67% of persons with chronic fatigue syndrome (CFS) and in 3.6% of healthy controls using PCR, serology, and virus isolation [5]. However, six subsequent studies found no association of XMRV and CFS in the US, Europe and China [6-11]. A more recent study failed to detect XMRV, but found a polytropic MuLV most similar to mouse endogenous retroviruses in 87% of CFS cases [12].

These discrepant results may be explained by differences in assay sensitivities used in each study, genetic heterogeneity of XMRV, geographic distribution of the virus, or by differences in subgroups of people with CFS. Since PCR assays have become standard tools in research and clinical laboratories, and each study reported using very sensitive assays, it is very unlikely that subtle assay differences contribute to these discordant test results. Some studies also used the same PCR assays as the initial study or generic tests for detecting both XMRV and other variants of MuLV [6-9], supporting further that the negative results were not due to assay differences or the ability to detect divergent viral strains.

The 1994 International Research Case Definition of CFS, currently used by most investigators, acknowledges that CFS subtypes are likely to occur, and encourages investigators to examine criteria to stratify cases, such as by type of onset, gradual or acute [11]. Variations in the approach to case ascertainment as well as in the severity of illness and type of onset could result in different spectrum of illness and potential differences in association with infection or other risk factors. It is also possible that the European studies [6-8] did not find XMRV due to regional differences or that the previous CDC study [9] was too localized to the regions around Georgia and in Wichita, Kansas. Similarly, a possible geographic clustering of XMRV infection has been observed in prostate cancer patients with most cases occurring in the US [2-4].

We tested fresh, EDTA-treated blood specimens from 30 CFS cases from 17 states in the US who consented to participate in a research study and who were recruited via an online announcement (Table 1). Blood was also collected from one additional person with CFS using heparin-containing collection tubes. Of these 31 persons, 26 were diagnosed by a doctor and 5 were self diagnosed. All CFS patients met the 1994 research case definition and specified a minimum of 6 months of post-exertional malaise and a high degree of disability, more closely resembling persons with CFS in the Lombardi *et al. *report than those CFS cases in previous studies. Specifically, we used Dr. Bell's CFS severity scale as an indicator of the degree of disability [13]. The mean low score experienced by our participants with "severe CFS" was 22.3, which is defined as "Moderate to severe symptoms at rest. Severe symptoms with any exercise; overall activity level reduced to 30%-50% of expected. Unable to leave house except rarely; confined to bed most of day; unable to concentrate for more than 1 hour a day" [13]. We also tested another 14 self-diagnosed CFS samples from persons having a severity score above 50 or having an unreported CFS severity (unclassified CFS) and 42 persons that did not have CFS. In total, samples came from more than 20 states, providing a broader geographic distribution than previous studies from the US (Table 1).

**Table 1:** Statistics on CFS patients and controls from the U.S

					Race		Gender	
**Population**	**N**	**States**	**AvgAge**	**Avg Duration of Illness**	**Caucasian**	**Other**	**Female**	**Male**

Severe CFS	31	17	44	12.8 yrs	87%	13%	61%	39%

Unclassified CFS	14	9	40	12.3 yrs	79%	21%	86%	14%

CFS Negative	42	12	23	n/a	71%	29%	45%	55%

**TOTAL**	**87**	**21**			**78%**	**22%**	**57%**	**43%**

Shows the number of states within the U.S. that participants were recruited from, the average participant age, the average time since the onset of CFS symptoms, the race and gender of participants from each class of sample.

Blood samples were shipped from collection centers overnight. Most were processed immediately upon arrival, but a few samples were incubated in the refrigerator for 1 to 2 days prior to separation of the blood components. For component separation, blood was centrifuged and the buffy coat, including the peripheral blood mononuclear cells (PBMCs), was immediately and carefully removed. The buffy coat was either processed immediately or stored at -20°C for later analysis. Nucleic acids were extracted using the Qiagen blood DNA minikit protocol (Qiagen, Valencia, CA). Extracted DNA was quantitated using the Nanodrop spectrophotometer (Thermo Scientific, Wilmington, DE) and checked for integrity with a minimum 260/280 ratio of 1.8 and by ß-actin PCR. Plasma was immediately frozen for later analysis.

**Figure 1:**
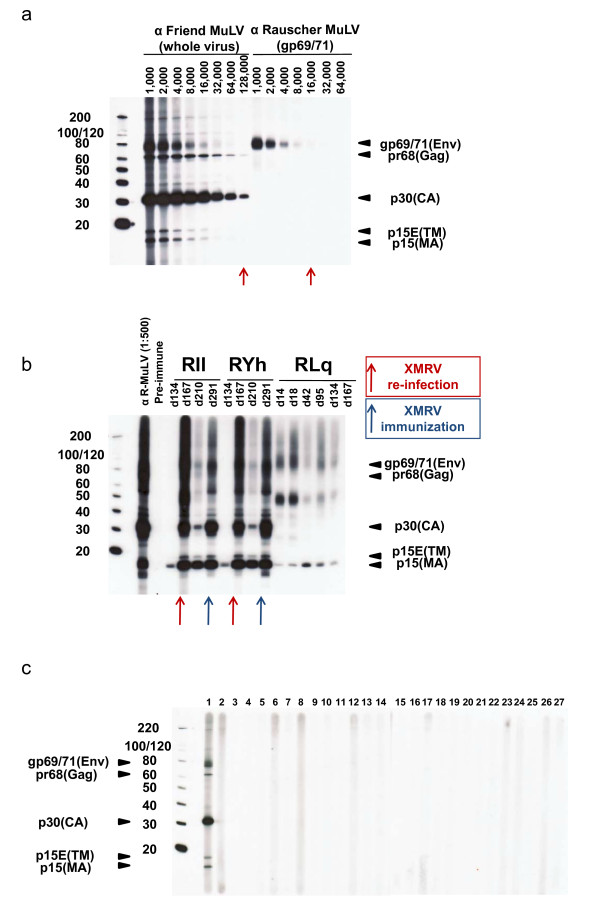
**Absence of antibodies to XMRV in plasma from persons with and without CFS from the US**. a. Antibody titers of positive control anti-sera to purified XMRV antigen in WB testing. Specific antisera tested are provided at the top of each WB. Arrows indicate observed titers for each antiserum. Locations of reactivity to specific viral proteins are indicated. Env (gp69/71), envelope; TM (p15E), transmembrane; MA (p15), matrix; Gag (pr68); CA (p30), capsid. Molecular weight markers (kD) are provided on the right of the WB. Sizes of expected viral proteins are provided to the left of the WB. b. Detection of XMRV antibodies in three experimentally-infected macaques (RII, RYh and RLq). Days post infection and immunization with XMRV are shown with arrows [16]. Locations of reactivity to specific viral proteins are indicated. Env (gp69/71), envelope; TM (p15E), transmembrane; MA (p15), matrix; Gag (pr68); CA (p30), capsid. Molecular weight markers (kD) are provided on the right of the WB. Sizes of expected viral proteins are provided to the left of the WB. c. Representative WB results for CFS cases and persons without CFS. Lane 1, 1:250 dilution of anti-Friend MuLV whole virus, goat polyclonal antisera; lane 2, XMRV negative blood donor plasma; lanes 3, 4, 9, 11 are plasma from persons without CFS; lanes 5 - 8, 10, 12, 14 - 17, 19, 20, 23 - 27 are plasma from persons with severe CFS; lanes 13, 18, 21, and 22 are plasma from persons with unclassified CFS. Locations of reactivity to specific viral proteins are indicated; Env (gp69/71), envelope; TM (p15E), transmembrane; MA (p15), matrix; Gag (pr68); CA (p30), capsid. Molecular weight markers (kD) are provided on the left of the WB.

PCR analysis was performed on PBMC DNA using three previously described tests (Table 2), two for the polymerase (*pol) *gene, and one for the *gag *gene used in Urisman *et al.*, Lombardi *et al*., and Lo *et al*. [1,5,9,14]. The *pol *real-time PCR test was used to analyze DNA samples from all study participants. At the CDC, nested *gag *(external primers GagOF and GagOR; internal primers GagIF and GagIR) and *pol *(external primers XPOLOF an XPOLOR; internal primers XPOLIF and XPOLIR) PCR was used to test a subset of specimens for which sufficient DNA remained, including 28 samples from "severe CFS" persons, 11 "unclassified CFS" and 9 controls [1,9]. 2.5 μg of DNA (833 ng of PBMC DNA) was used in the *pol *real-time PCR test, providing for 3.3 to 8.3 times the PBMC DNA used by Lombardi *et al*. [5,14]. Dilutions of DNA from XMRV-infected 22Rv1 human prostate carcinoma cells were used as positive controls in this test [15]. 1.0 μg of DNA (333 ng of PBMC DNA) was used in the nested *pol *and *gag *PCR tests at the CDC for which 1,000 and 10 copies of the XMRV(VP62) plasmid were used as positive controls [1,9]. A subset of 48 plasma samples were tested for viral RNA sequences by RT-PCR using primers from the nested *gag *assay and also by using a new quantitative RT-PCR test that generically detects MuLV and XMRV *gag *sequences. Both RT-PCR tests could detect between 10 - 25 copies of XMRV (VP62) RNA. Since antibody responses are hallmarks of retroviral infection, we also used a newly modified Western blot (WB) test to detect anti-XMRV antibodies in plasma [1,9]. Serologic tests could potentially also identify low-level or latent XMRV infection not otherwise detectable by PCR. Briefly, XMRV-infected DU145 prostate cells (C7) were grown in complete HuMEC serum free medium supplemented with 1% HuMEC and 50 ug/ml bovine pituitary extract (Invitrogen). Tissue culture supernatants were clarified by centrifugation and by passage through a 0.45 um filter. XMRV was purified from 150 ml C7 supernatant using the ViraTrap Retrovirus Maxiprep Kit (Bioland Scientific LLC) following the manufacturer's protocol. 150 ul of purified XMRV was denatured with SDS-PAGE sample buffer at 95°C for 10 minutes, and viral proteins were separated by gel electrophoresis in a NuPAGE 4-12% Bis-Tris gel (Invitrogen) for WB testing as previously described but modified by using horseradish peroxidase conjugated protein G instead of protein A/G [9]. Seroreactivity was defined by reactivity to viral Env and/or Gag proteins of the expected size as seen in the positive control antisera (Figure 1). This new WB test accurately detects XMRV antibodies in three experimentally infected macaques equivalent to detection using recombinant proteins in recently described immunoassays (Figure 1b) [16]. All PCR and WB testing at the CDC were performed blinded to diagnosis.

**Table 2:** PCR oligos and conditions

Oligo Name	Sequence (5'→3')	**Location**^**1**^	Sample	Conditions
**pol Forward**	GGGGATCAAGCCCCACATA	2794 to 3062	2.5 μg DNA	95°C for 20 s followed by 45 cycles of 95°C for 1 s and 60°C for 20 s [14]
**Reverse**	GGTGGAGTCTCAGGCAGAAAA			
**Probe**	[6FAM] TGTTCCAGGGGGACT GGCAAGGTACCAccctgg [DABC]^2,3^			

**pol2 XPOLOF**	CCGTGCCCAACCCTTACAACCTCT	2961 to 3330	1.0 μg DNA	40 cycles of 94°C for 30 s, 50°C for 30 s, 72°C for 45 s for both primary and nested PCR [9]
**XPOLOR**	CCGAGGTTCCCTAGGGTTTGTAAT			
**XPOLIF**	TCCACCCCACCAGTCAGCCTCTCT			
**XPOLIR**	AAGTGGCGGCCAGCAGTAAGTCAT			
**XPOLP**	TTGATGAGGCACTGCACAGAGACC	Probe		

**gag1 GagOF**	ATCAGTTAACCTACCCGAGTCGGAC	419 to 1149	0.25 μg DNA; RNA from 62 μL plasma	40 cycles of 94°C for 30 s, 50°C for 30 s, 72°C for 45 s for both primary and nested DNA PCR [5,9]. RT-PCR; Primer 1154R was used for cDNA synthesis at 42°C for 1 hr with the IScript Select cDNA kit (BioRad) followed by 85°C, 5 min to stop the reaction. Nested PCR was then performed as for DNA testing using the Expand High Fidelity PCR System (Roche) and AmpliTaq (Applied Biosystems) for the primary and nested PCRs.
**GagOR**	GCCGCCTCTTCTTCATTGTTCTC			
**GagIF**	GGGGACGAGAGACAGAGACA			
**GagOR**	CAGAGGAGGAAGGTTGTGCT			
**XGagP2**	ACCTTGCAGCACTGGGGAGATGTC	Probe		

**gag2 Forward**	AGGTAGGAACCACCTAGTYC	1581 to 1764	RNA from 62 μL plasma	RT-PCR using AgPath-ID one step RT-PCR kit (Applied Biosystems) and BioRad iQ5 iCycler. Reverse primer used for cDNA synthesis at 45°C for 20 min; 95°C for 10 min. 55 cycles at 95°C, 30 s, 52°C, 30 s, 62°C, 30 s.
**Reverse**	GTCCTCAGGGTCATAAGGAG			
**Probe F**	[6FAM]AGCGGGTCTCCAAAACGCGGGC[BHQ1]^3^	1620		
**Probe R**	[6FAM]CCTTTTACCTTGGCCAAATTGGTGGGG[BHQ1]^3^	1673		

^1^Reference sequence was the VP62 XMRV strain (GenBank: EF185282.1).

^2^Lower case bases were added to form the stem.

^3^[6FAM] and [DABC] and [BHQ1] are the fluorophore FAM and the quenchers Dabcyl and Blackhole, respectively.

**Figure 2:**
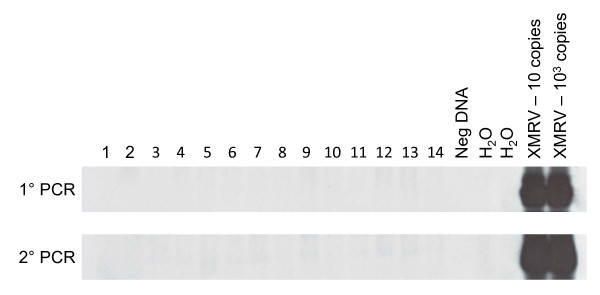
**Absence of XMRV/MuLV sequences by PCR of PBMC DNA of persons with and without CFS from the US**. Representative nested polymerase (*pol*2) PCR results. Lanes 1 and 2 are results from persons without CFS; lanes 3 - 8, 10 and 11 are results from patients classified with severe CFS; lanes 9, and 12 - 14 are results from patients with unclassified CFS; lane 15, negative human PBMC DNA control; lanes 16 and 17, water only controls; lanes 18 and 19, assay sensitivity controls consisting of 10^1 ^and 10^3 ^copies of XMRV VP62 plasmid DNA diluted in a background of 1 μg of human PBMC DNA, respectively.

**Figure 3:**
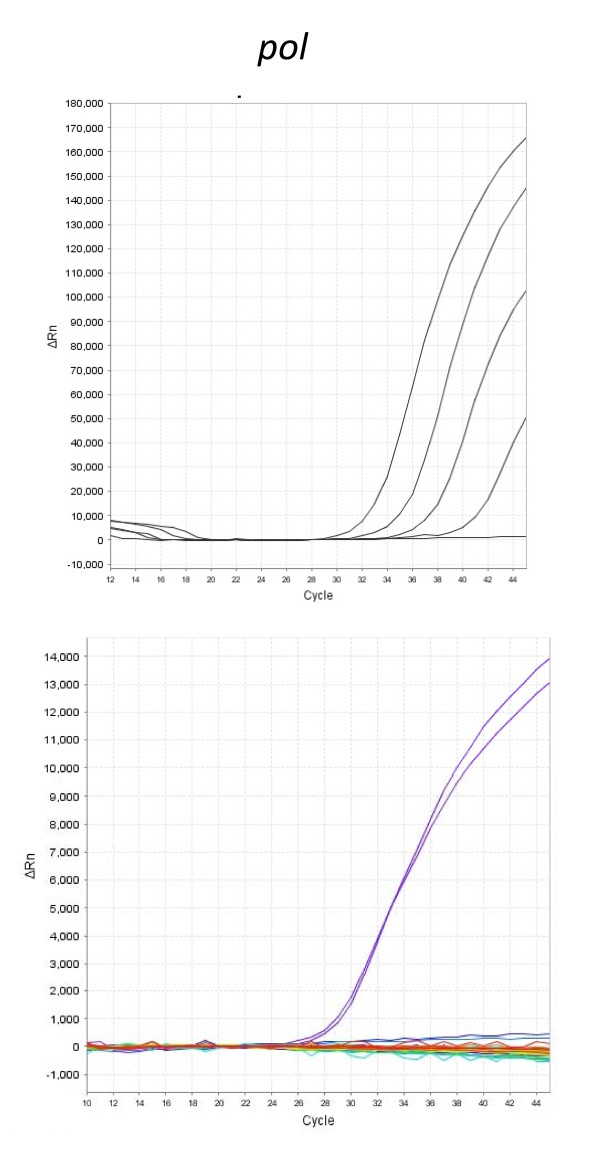
**Absence of XMRV/MuLV sequences by real-time PCR in PBMC DNA of persons with and without CFS from the US**. Representative real-time XMRV polymerase (*pol) *PCR results. **Upper panel; ***pol *amplification plot using XMRV synthetic DNA diluted in a background of 2.5 ug of DNA from whole blood to 12,000, 1,200, 120 and 12 copies and negative (water and DNA) controls demonstrating the sensitivity and dynamic linear range of the assay. **Lower panel; ***pol *amplification plot for DNA from 40 persons, including 18 with severe CFS, 8 with unclassified CFS, and 14 without CFS. Two positive controls (DNA from 17 XMRV infected 22Rv1 cells spiked into 2.5 μg of human leukocyte DNA) for the *pol *PCR, and two negative controls (2.5 μg of DNA) are also shown. Only the two positive controls were detected in this testing.

Using this comprehensive testing strategy to test CFS samples from persons with post-exertional malaise from a variety of US states, we did not find any serologic or molecular evidence of XMRV or MuLV in persons with or without CFS (Table 3, Figures 1, 2 and 3). These results suggest that neither the limited geographic locality of previous publications nor the post-exertional malaise criteria explain the discrepant results seen in previous studies.

**Table 3:** Absence of XMRV in CFS patients from the U.S

		XMRV Positive					
**Population**	**N**	***pol***	***pol*2**	***gag1***	**WB**	***gag1*** **RT-PCR**	***gag*2** **RT-PCR**

Severe CFS	31	0/31	0/28	0/28	0/28	0/28	0/28

Unclassified CFS	14	0/14	0/11	0/11	0/11	0/11	0/11

CFS Negative	42	0/42	0/9	0/9	0/9	0/9	0/9

**TOTAL**	**87**	**0/87**	**0/48**	**0/48**	**0/48**	**0/48**	**0/48**

Cooperative Diagnostics *pol *real-time PCR test, CDC *pol *(*pol2*) and *gag *DNA PCR, *gag *RT-PCR, and Western blot (WB) results.

For detection of any new virus, false positive and negative results are always a concern, especially when bona fide positive and negative clinical specimens are not available for assay validation. The PCR tests in this study have been previously shown to detect low levels (≤ 10 copies) of XMRV plasmid in high genomic DNA backgrounds and are capable of generically detecting XMRV and diverse MuLVs [5,9,14]. While all the PCR tests used in XMRV studies reported similar sensitivities, it is important to note that each used a different amount of starting DNA. Specifically, the assays of Lo *et al*. and Lombardi *et al. *can at best detect 1 copy of XMRV/MuLV in a background of 30 to 50 ng and 100 to 250 ng of DNA respectively [5,12]. However, in our study, we use the most sensitive PCR test reported to date, with a detection limit of 1 copy of XMRV or MuLV in 2,500 ng of DNA, a 10-83X improved detection limit over the assays used by Lombardi *et al. *and Lo *et al*. This indicates that any one of the assays would be able to detect XMRV or MuLV if present in the samples. Moreover, a recent study also demonstrated the importance of using at least 600 ng of input DNA to increase detection of XMRV in prostate cancer patients [17]. XMRV could also be present in blood at levels below the detection limit of PCR, but this seems unlikely given the relatively high frequency of infection reported by Lombardi *et al*. and Lo *et al. *in people with CFS using tests with less sensitive PCR tests [5,12]. Unlike other reports [5,12], we also found no evidence of active XMRV/MuLV viremia using highly sensitive RT-PCR tests excluding possibilities of peripheral infection seeding the blood compartment from other body locations. Furthermore, WB testing did not detect XMRV or MuLV antibodies in the plasma samples, arguing against the development of an XMRV/MuLV-specific humoral immune response, as is commonly seen with other human retroviral infections, and precluding the possibility of low level viral infection in blood or in other reservoirs. Given the recent finding that an XMRV antibody test, using even a single XMRV protein, had 100% sensitivity for XMRV detection in monkeys after the second week of infection with XMRV, it is highly unlikely that our WB test, which uses purified, whole XMRV as antigen and detects XMRV antibodies in infected macaques, would have missed detecting XMRV infection [16].

It is also important to note that the report by Lo *et al*. is not a confirmation of the Lombardi *et al*. study since like previous studies, this study also failed to identify XMRV in any of the CFS samples or controls [6-12]. Rather, Lo *et al*. identified a polytropic MuLV sequence in a majority of CFS samples that most closely resembles nonfunctional viruses in mouse genomic DNA, which was confirmed by a truncated Gag sequence in one CFS specimen in their study. Thus, without viral isolation or complete genomes, the infectivity and person-to-person transmissibility of these polytropic viruses are unclear. Others have described the lengthy history and ubiquitous nature of mouse cell or DNA contamination, even in laboratories that have never worked with MuLV's, and concluded that contamination cannot be excluded as a source of the MuLV-like sequences in some studies [18]. Since this report, four laboratories have reported that 100% of polytropic MuLV and/or XMRV sequences found in their CFS and prostate cancer samples stemmed from contamination from commercial reagents and/or other sources [11,19-21]. In addition, a review on XMRV describes the potential dangers from using polymerases with antibody mediated hot starts, especially those developed from mouse hybridoma cells, such as the Platinum Taq used by Lo *et al. *[22]. While Lo *et al. *did not find mouse cell contamination by a retrospective screen of their samples for murine mitochondrial sequences or through the use of numerous water controls, mtDNA screening and water controls are not sufficient to detect the majority of murine genomic DNA contamination [19,20]. Hue *et al*. showed that 100% of published XMRV sequences from CFS and prostate cancer samples have less sequence variation than occurs within XMRV in the 22Rv1 cell line, concluding that any discovery of these conserved XMRV sequences in patient samples was due to contamination [23]. Given the high degree of known risk for contamination even in laboratories that have never worked with MuLV's and the historical contamination of human cell lines with MuLVs and other retroviruses [18,24], it is imperative that murine contamination controls be run in parallel with all human testing. Since both polytropic and xenotropic MuLV's are capable of infecting non-murine cells, other controls will need to be developed to rule out contamination from non-murine sources.

In conclusion, we have used a comprehensive testing strategy, including highly sensitive PCR tests and a novel XMRV WB assay, to show that neither the limited geographic differences of previous studies within the United States nor the condition of post-exertional malaise are the reason for the discordant study results. Further, with what are now seven negative studies, it is highly unlikely that XMRV is present in people with CFS or in control populations as frequently as has been previously reported. The amount of specimen from each of the positive studies has been limiting for independent confirmation of the test results. Thus, different study designs are needed to further investigate an association of XMRV and MuLV in persons with CFS, including carefully defined case control studies in which specimens are collected and processed the same, followed by coded and blinded testing at independent laboratories reporting both detection and absence of infection with these viruses.

## Competing interests

Cooperative Diagnostics is a commercial enterprise that owns the rights to one of the XMRV PCR tests described in this manuscript. Publication of these results will likely reduce the potential market that Cooperative Diagnostics could reach with its XMRV test. The findings and conclusions in this report are those of the authors and do not necessarily represent the views of the Centers for Disease Control and Prevention.

## Authors' contributions

BCS and WMS planned and conceived the experiments and analyzed the results. RAG, ST, HZ and HJ performed the tests and analyzed the data. HJ developed the XMRV WB test. HZ developed the gag qRT-PCR test. BCS and WMS wrote the paper. All authors read and approved the final manuscript.
